# Metabolite profiling of guanfacine in plasma and urine of healthy Japanese subjects after oral administration of guanfacine extended‐release tablets

**DOI:** 10.1002/bdd.2201

**Published:** 2019-08-07

**Authors:** Yuji Inoue, Hirotoshi Morita, Kohei Nozawa, Takushi Kanazu

**Affiliations:** ^1^ Drug Metabolism & Pharmacokinetics Shionogi & Co., Ltd., Toyonaka Osaka Japan; ^2^ Analytical Chemistry & Bioanalysis Shionogi TechnoAdvance Research Co., Ltd., Toyonaka Osaka Japan; ^3^ Drug Development Solutions Center Sekisui Medical Co., Ltd., Tokai Ibaraki Japan

**Keywords:** ADHD, guanfacine extended release, human, LC–MS/MS, metabolic pathway

## Abstract

Guanfacine is used for the treatment of attention‐deficit/hyperactivity disorder (ADHD). Using liquid chromatography–tandem mass spectrometry (LC–MS/MS), metabolite profiling of guanfacine was performed in plasma and urine collected from healthy Japanese adults following repeated oral administration of guanfacine extended‐release formulation. Unchanged guanfacine was the most abundant component in both plasma and urine (from the MS signal intensity). In plasma, the M3 metabolite (a sulfate of hydroxy‐guanfacine) was the prominent metabolite; the M2 metabolite (a glucuronide of a metabolite formed by monooxidation of guanfacine), 3‐hydroxyguanfacine and several types of glucuronide at different positions on guanfacine were also detected. In urine, the M2 metabolite and 3‐hydroxyguanfacine were the principal metabolites. From metabolite analysis, the proposed main metabolic pathway of guanfacine is monooxidation on the dichlorobenzyl moiety, followed by glucuronidation or sulfation. A minor pathway is glucuronidation at different positions on guanfacine. As the prominent metabolites in plasma were glucuronide and sulfate of hydroxyguanfacine, which have no associated toxicity concerns, further toxicity studies of the metabolites, for example in animals, were not deemed necessary.

## 1 | INTRODUCTION

Attention‐deficit/hyperactivity disorder (ADHD) is a neurobehavioral condition, predominantly identified during childhood and characterized by various degrees of developmentally inappropriate inattention, hyperactivity and impulsiveness (Goldman, Genel, Bezman, & Slanetz, 1998). In Japan, only two medications are available for the treatment of ADHD. The first, indicated for pediatric ADHD, is an extended‐release formulation of methylphenidate hydrochloride (Concerta^®^, Janssen Pharmaceutical K.K., Shizuoka, Japan), which was approved in 2007 as a psychostimulant drug. The second, approved in 2009, is atomoxetine hydrochloride (Strattera^®^, Eli Lilly Japan K.K., Kobe City, Japan), classified as a non‐psychostimulant drug. Additional medicine approvals for the management of ADHD will provide Japanese patients and clinicians with more treatment options. Guanfacine is a selective α_2_‐adrenergic receptor agonist (Huss, Chen, & Ludolph, 2016), which is a centrally acting agent that stimulates α_2A_‐adrenergic receptors. Its activity is closely related to an increase in noradrenaline activity. An extended‐release formulation of guanfacine (guanfacine XR; Intuniv^®^ [Shire LLC, Lexington, MA, USA]) received approval for the treatment of ADHD in the United States in 2009 (FDA, 2009) and in Europe in 2015 (EMA, 2015) and was approved for the treatment of pediatric ADHD in Japan in 2017 (PMDA Japan, 2017).

Metabolite profiling and the identification of guanfacine in human subjects has previously been conducted with urine samples (Kiechel, 1980). However, metabolite profiling and identification in human plasma samples has not been conducted. The guidelines of the International Council for Harmonisation of Technical Requirements for Pharmaceuticals for Human Use (ICH) M3(R2) (ICH, 2009, 2012) require nonclinical characterization of a human metabolite(s) when the metabolite(s) comprises more than 10% of the measured total exposure to drug and metabolites, usually based on the group mean area under the plasma concentration–time curve (e.g. AUC from zero to infinity), and when the metabolite(s) is (are) found at significantly greater levels in humans than the maximum exposure observed in toxicity studies in animals. Data on *in vivo* metabolism in the test species and humans should be available before exposing large numbers of human subjects or to a long duration of exposure (generally before Phase 3).

Therefore, this Phase 1 study was conducted to elucidate the prominent metabolite(s) in the plasma and urine of healthy adult Japanese subjects after multiple doses of guanfacine XR. Furthermore, possible metabolic pathways of guanfacine based on *in vivo* metabolites found in humans are shown.

## 2 | MATERIALS AND METHODS

### 2.1 | Materials

Guanfacine XR tablets (4 mg) were supplied by DSM Pharmaceuticals Inc. (Greenville, SC, USA). Reference standards for metabolite analysis of guanfacine (S‐877503, purity > 98% by high‐performance liquid chromatography [HPLC]) and 3‐hydroxyguanfacine (purity > 95% by HPLC) were supplied by Shionogi & Co., Ltd. The reagents (formic acid, methanol and distilled water) were of guaranteed liquid chromatography‐mass spectrometry (LC–MS) grade, and the reagents (ammonium acetate and acetonitrile) were of guaranteed grade.

### 2.2 | Samples for metabolite analysis

The samples (plasma and urine) for metabolite analysis used in this study were obtained in a Phase 1 pharmacokinetics study of single and multiple doses of S‐877503 in healthy Japanese and Caucasian subjects (Matsuo, Okita, Ermer, & Wajima, 2017). The clinical study was approved by an institutional review board (P‐One Clinic, Keikokai Medical Corporation) and was conducted in accordance with the ICH Good Clinical Practice guidelines, the principles of the Declaration of Helsinki, and any other applicable local ethical and legal requirements; all subjects gave written, informed consent (Matsuo et al., 2017). A total of 12 healthy Japanese male subjects aged between 20 and 44 years received multiple oral doses of guanfacine XR at 1 mg (days 1–5), 2 mg (days 6–10), 3 mg (days 11–15) and 4 mg (days 16–20) in ascending dose, once daily for 5 consecutive days at each dose. Samples from 11 subjects were used for metabolite analysis, as one subject withdrew from the study for personal reasons. Blood was collected to obtain plasma for metabolite analysis at predose (before 1 mg dosing), and at 2, 4, 6, 10 and 24 hours after the final dosing of 4 mg treatment. Urine samples were collected over 12 hours predose (before 1 mg dosing), and over 0–12 and 12–24 hours after final dosing of the 4 mg treatment. All samples were stored at approximately −70 °C and protected from light until analysis.

### 2.3 | Metabolite analysis

Each sample of plasma and urine was prepared by pooling with the same volume of samples collected from the 11 actively administered subjects at each time point. One ml of pooled plasma samples was mixed with 3 ml of 0.1% formic acid in acetonitrile as the extraction solution, shaken (10 min) and centrifuged (1800 × *g*, 4 °C, 10 min) to separate the supernatant. Residues were extracted twice with the same volume of extraction solution in the same manner as the first extraction. All obtained supernatants were combined and evaporated to dryness under reduced pressure, and the residues were reconstituted in 300 μl of reconstitution solution, which consisted of 10 mmol/l ammonium acetate in 0.2% formic acid aqueous solution and 0.2% formic acid in methanol (80:20, v/v). The reconstituted samples were centrifuged (1800 × *g*, 4 °C, 5 min), and the supernatants were used as the samples for metabolite analysis.

An aliquot of 100 μl of pooled urine samples was mixed with 10 μl of 0.1% formic acid and centrifuged (1800 × *g*, 4 °C, 10 min). The supernatant was then used as the sample for metabolite analysis. The metabolites produced from guanfacine were numbered in order of retention times (RTs; M1 to M9).

### 2.4 | Composition ratio of metabolites

The composition ratio of the metabolites was calculated by taking the ratio of the peak area of each metabolite component to the total peak area of all detected metabolite components in each sample. For metabolites where standards were not available, their extraction recovery, reconstitution recovery and MS response (ionization efficiency) were assumed to be unity (1).

$$\text{Total}\;\left(\%\right)=\left[\text{Peak area of each component}\right]/\left[\text{Total peak area of}\;\mathrm{all}\;\text{components}\right]\times 100$$

### 2.5 | LC–MS/MS equipment

All analyses were performed using a HPLC system (LC‐20A; Shimadzu, Kyoto, Japan) equipped with a mass spectrometer (LTQ Orbitrap XL; Thermo Fisher Scientific, Waltham, MA, USA). An XBridge C18 column (3.5 μm, 2.1 × 150 mm; Waters, Santa Clara, CA, USA) and Security Guard Cartridge C18 column (4 × 3.0 mm; Phenomenex, Torrance, CA, USA) were used for HPLC with tandem mass spectrometry (LC–MS/MS) analysis. The column temperature was maintained at 40 °C and the mobile phase consisted of a mixture of 10 mmol/l ammonium acetate in 0.2% formic acid aqueous solution (A) and 0.2% formic acid in methanol (B). The solvent gradient conditions were: 0 min, 5% B; 0–30 min, 5–50% B; 30–30.1 min, 50–98% B; 30.1–33 min, 98% B; 33–33.1 min, 98–5% B; and 33.1–45 min, 5% B. The flow rate was set at 0.3 ml/min. Mass spectra were obtained in the positive ion mode and the ionization interface was electrospray ionization (ESI). Optimized parameters were set as follows: the spray voltage was 4.0 kV, the capillary temperature was 350 °C, the tube lens voltage was 45 V, the sheath gas was 50 arb (N_2_), the auxiliary gas was 20 arb (N_2_), the sweep gas was 3 arb (N_2_), the collision gas was He, the normalized collision energy was 35% (collision‐induced dissociation [CID]) and the isolation width was 2 u. All data were obtained and processed using Xcalibur software (Thermo Fisher Scientific, Waltham, MA, USA).

## 3 | RESULTS

### 3.1 | Metabolite analysis of plasma and urine samples

Guanfacine, 3‐hydroxyguanfacine, M1 to M4, M7 and M8 were detected in plasma samples using LC–MS/MS (Table 1). Guanfacine was the most abundant component and M3 was the most prominent metabolite, followed by M2.

**Table 1:** Guanfacine and its metabolites in human plasma and urine

Metabolite	Retention time (min)	Molecular formula [M + H]^+^	Mass shift (Da)	Plasma	Urine
M1	3.7–4.0	C_9_H_12_Cl_2_N_3_O_3_	+34	Detected	Detected
M2	10.6–10.8	C_15_H_18_Cl_2_N_3_O_8_	+192	Detected	Detected
M3	13.3–13.4	C_9_H_10_Cl_2_N_3_O_5_S	+96	Detected	Detected
3‐Hydroxyguanfacine	16.0	C_9_H_10_Cl_2_N_3_O_2_	+16	Detected	Detected
M4	16.3–16.4	C_15_H_18_Cl_2_N_3_O_7_	+176	Detected	Detected
M5	16.8	C_15_H_18_Cl_2_N_3_O_7_	+176	−	Detected
M6	17.2	C_9_H_10_Cl_2_N_3_O_2_	+16	−	Detected
M7	20.6–20.7	C_15_H_18_Cl_2_N_3_O_7_	+176	Detected	Detected
M8	22.0	C_15_H_18_Cl_2_N_3_O_7_	+176	Detected	Detected
Guanfacine	22.4	C_9_H_10_Cl_2_N_3_O	−	Detected	Detected
M9	25.0	C_14_H_18_Cl_2_NO_8_	+152	−	Detected

Guanfacine, 3‐hydroxyguanfacine and M1 to M9 were detected in urine samples (Table 1). Guanfacine was the most abundant component, followed by M2 and 3‐hydroxyguanfacine. In both plasma and urine, there were no apparent differences in the composition ratio of the metabolites at each sampling point, suggesting that the elimination of the metabolites was not changed.

### 3.2 | LC–MS (MS)^n^ measurement of authentic standards

**Figure 1:**
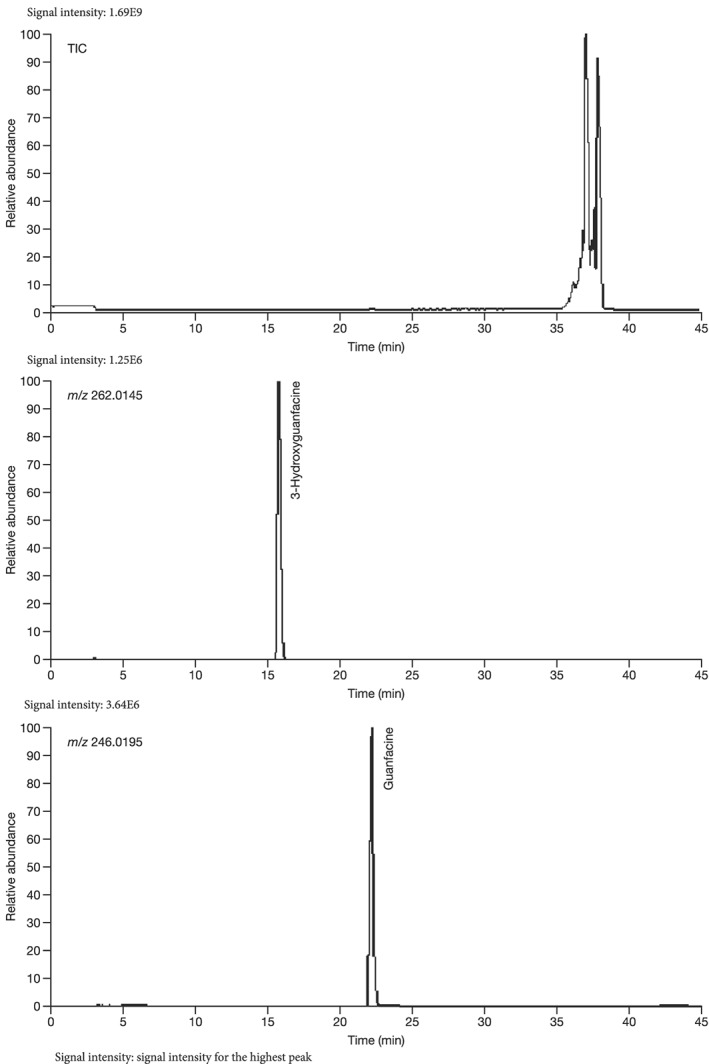
LC/ESI (+) MS chromatograms of reference standards. TIC, total ion chromatogram

**Figure 2:**
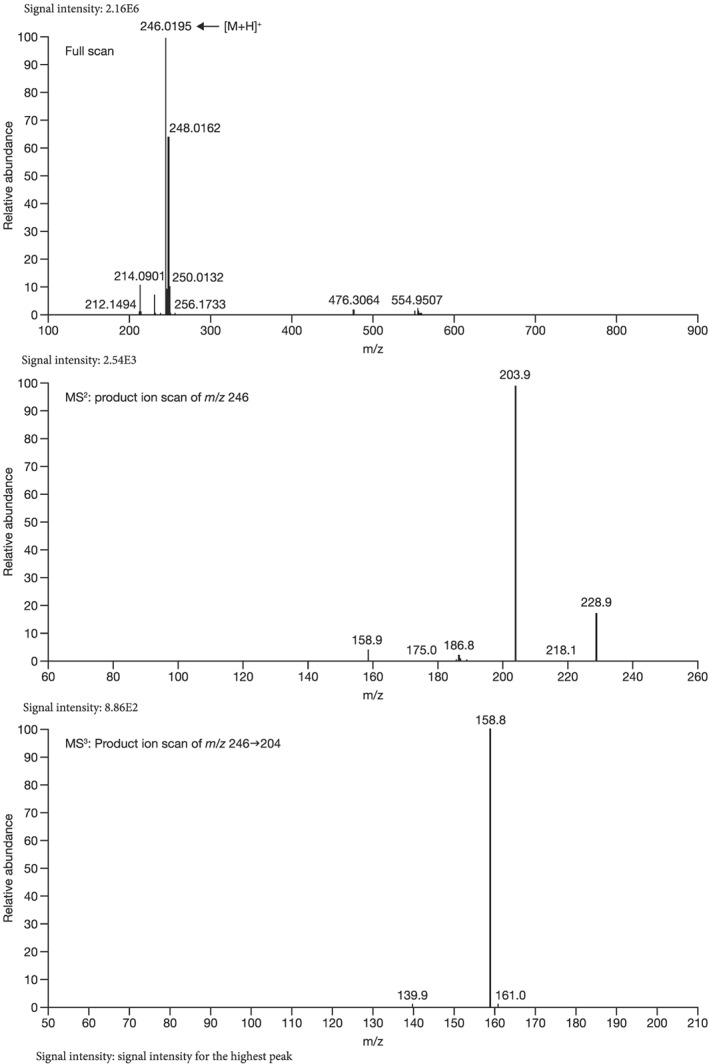
LC/ESI (+) MS and MS^n^ spectra of guanfacine. [M + H]^+^, protonated molecular ion

**Figure 3:**
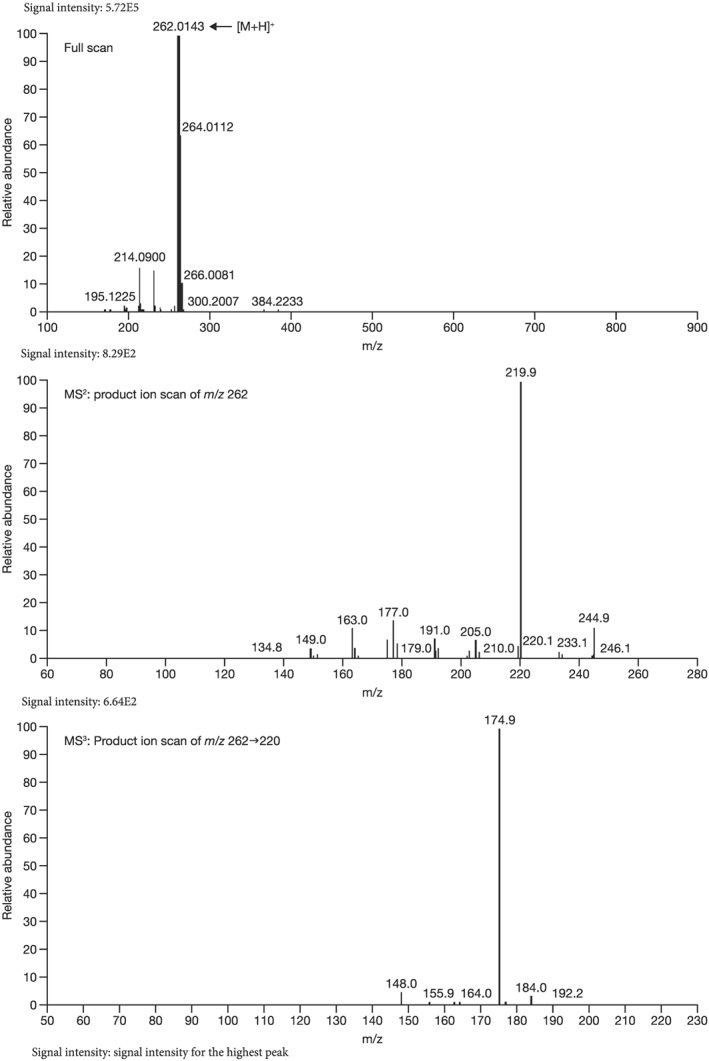
LC/ESI (+) MS and MS^n^ spectra of 3‐hydroxyguanfacine. [M + H]^+^, protonated molecular ion

Mass spectrometry (MS) chromatograms of authentic standards are shown in Figure 1. MS and MS^n^ spectra of authentic standards are shown in Figure 2 (guanfacine) and Figure 3 (3‐hydroxyguanfacine).

Guanfacine was found at a RT of approximately 22 min with ESI in the positive ion mode at protonated molecular ion [M + H]^+^ of *m/z* 246 (Figure 1). MS^2^ and MS^3^ fragment ions were observed at *m/z* 204 (−42) and 159 (204–45) (Figure 2). The MS^2^ fragment ion at *m/z* 204 was estimated to be formed by degradation of the guanidine group. Subsequently, the MS^3^ fragment ion observed at *m/z* 159 from *m/z* 204 was considered to be due to further elimination of the amide unit. The structure of the fragment ion at *m/z* 159 was postulated to be the 2,6‐dichlorobenzyl cation.

3‐Hydroxyguanfacine was found at the RT of approximately 16 min with *m/z* 262 (Figure 1). MS^2^ and MS^3^ fragment ions were observed at *m/z* 220 (−42) and 175 (220–45) (Figure 3). The fragmentation of 3‐hydroxyguanfacine was equivalent to guanfacine. The structure of the fragment ion at *m/z* 175 was postulated to be the 2,6‐dichloro‐3‐hydroxybenzyl cation.

### 3.3 | Structural identification

**Figure 4:**
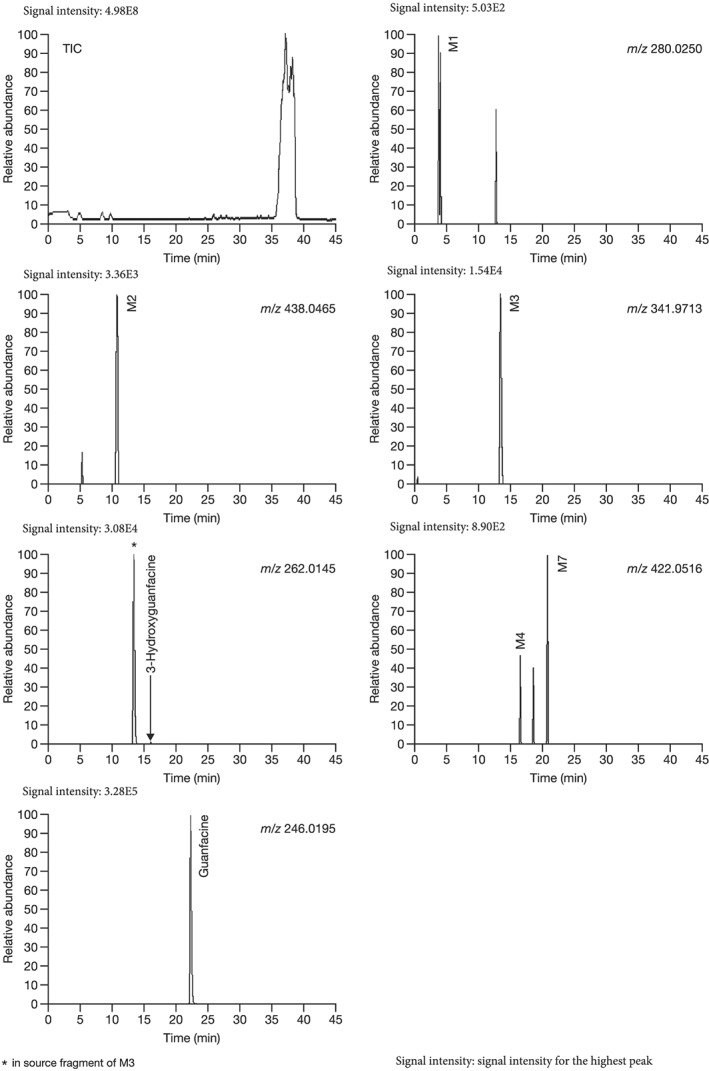
LC/ESI (+) MS chromatograms of guanfacine and its metabolites (M) in human plasma, 6 hours after final dosing. TIC, total ion chromatogram

**Figure 5:**
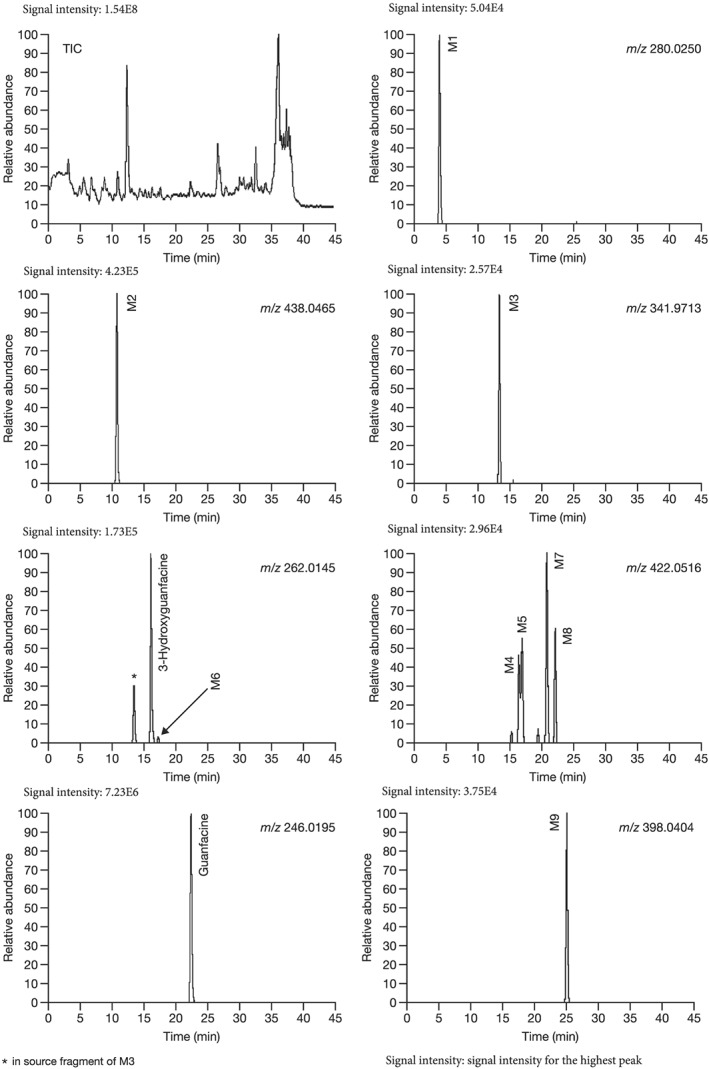
LC/ESI (+) MS chromatograms of guanfacine and its metabolites (M) in human urine, 12–24 hours after final dosing. TIC, total ion chromatogram

The postulated structures and key fragment ions of guanfacine and its metabolites (3‐hydroxyguanfacine, M1–M9) are shown in Table 2. Representative MS chromatograms of guanfacine and its metabolites are shown in Figure 4 (plasma) and Figure 5 (urine). The structural rationalization of each metabolite is explained in detail below.

**Table 2:** Postulated structures of guanfacine metabolites in human plasma and urine

Metabolite	*m/z*	Structure	Key fragment ions
Guanfacine	246	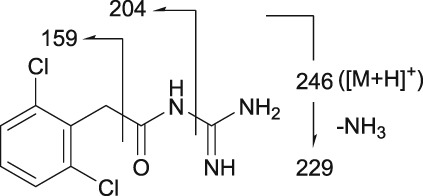	159, 204, 229, 246
3‐Hydroxyguanfacine	262	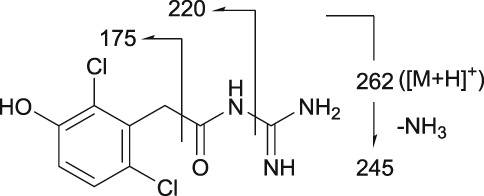	175, 220, 245, 262
M1	280	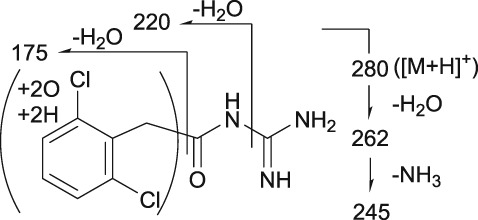	175, 220, 245, 262, 280
M2	438	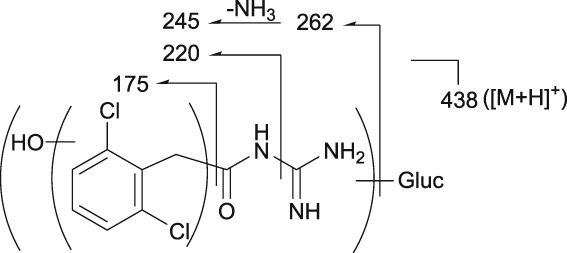	175, 220, 245, 262, 438
M3	342	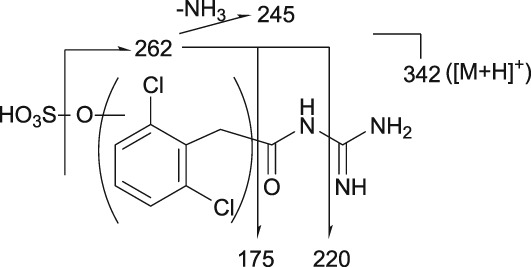	175, 220, 245, 262, 342
M4	422	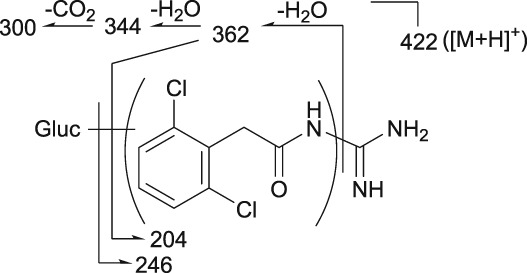	204, 246, 300, 344, 362, 422
M5	422	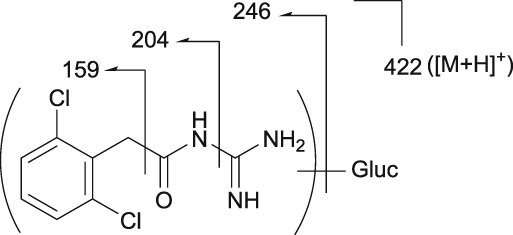	159, 204, 246, 422
M6	262	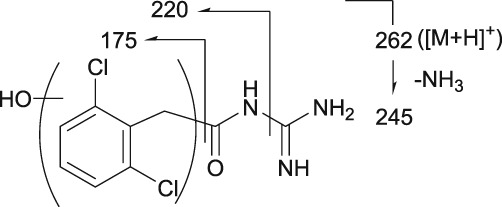	175, 220, 245, 262
M7	422	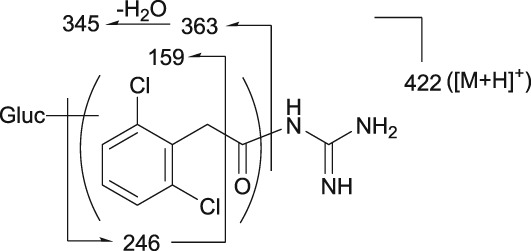	159, 246, 345, 363, 422
M8	422	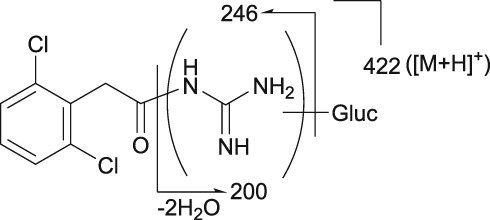	200, 246, 422
M9	398	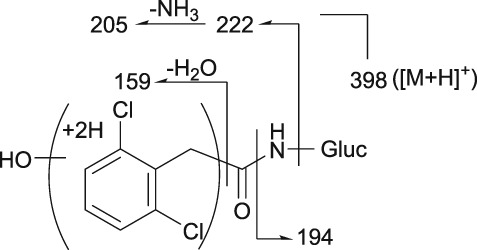	159, 194, 205, 222, 398

[M + H]^+^, protonated molecular ion; Glu, glucuronic acid.

### 3.4 | Guanfacine

The fragment ion at *m/z* 246 was detected at the same RT as guanfacine in urine at 0–12 and 12–24 hours, and in plasma at 2, 4, 6, 10 and 24 hours (Figures 4, 5). The estimated molecular formula of C_9_H_10_Cl_2_N_3_O was equivalent to guanfacine, and the resulting MS^2^ and MS^3^ fragment ions at *m/z* 204 and 159 were also equivalent to the authentic standard (Table 2 and Figure 2). Therefore, this component was identified as guanfacine.

### 3.5 | 3‐Hydroxyguanfacine

The fragment ion at *m/z* 262 was detected at the same RT as 3‐hydroxyguanfacine in urine at 0–12 and 12–24 hours, and in plasma at 6 hours (Figures 4, 5). The estimated molecular formula of C_9_H_10_Cl_2_N_3_O_2_ was equivalent to 3‐hydroxyguanfacine. Moreover, the resulting MS^2^ and MS^3^ fragment ions at *m/z* 220 and 175 were also equivalent to the authentic standard (Table 2 and Figure 3). Therefore, this component was identified as 3‐hydroxyguanfacine.

### 3.6 | M1 metabolite

M1 was detected at the RT of approximately 4 min with *m/z* 280 in urine at 0–12 and 12–24 hours, and in plasma at 2, 4, 6 and 10 hours (Figures 4, 5). In the MS^2^ spectrum, *m/z* 220 was estimated to be generated by degradation of the guanidine group and dehydration. The MS^2^ product ion at *m/z* 175 was postulated to be a monooxidized 2,6‐dichlorobenzyl cation, which was estimated to be generated by dehydration of its corresponding moiety (Table 2). Given these results, M1 was elucidated to be a metabolite formed by dioxidation and hydrogenation of the dichlorobenzyl moiety of guanfacine.

### 3.7 | M2 metabolite

M2 was detected at the RT of approximately 11 min with *m/z* 438 in urine at 0–12 and 12–24 hours, and in plasma at 2, 4, 6, 10 and 24 hours (Figures 4, 5). The MS^2^ product ion at *m/z* 262, which was estimated to be an aglycone of the M2, generated MS^3^ product ion at *m/z* 220 and MS^4^ product ion at *m/z* 175 (Table 2). These subsequent fragmentations were equivalent to authentic 3‐hydroxyguanfacine; however, the position of hydroxylation in the dichlorobenzyl moiety could not be assigned. Given these results, M2 was elucidated to be a glucuronide of a metabolite formed by monooxidation of the dichlorobenzyl moiety.

### 3.8 | M3 metabolite

M3 was detected at the RT of approximately 13 min with *m/z* 342 in urine at 0–12 and 12–24 hours, and in plasma at 2, 4, 6, 10 and 24 hours (Figures 4, 5). The MS^2^ product ion at *m/z* 262, which was estimated to be an aglycone of the M3, generated MS^3^ product ion at *m/z* 220 and MS^4^ product ion at *m/z* 175 (Table 2). These subsequent fragmentations were equivalent to those of authentic 3‐hydroxyguanfacine; however, the position of hydroxylation in the dichlorobenzyl moiety could not be assigned. Given these results, M3 was elucidated to be a sulfate of a metabolite formed by monooxidation of the dichlorobenzyl moiety.

### 3.9 | M4 metabolite

M4 was detected at the RT of approximately 16 min with *m/z* 422 in urine at 0–12 and 12–24 hours, and in plasma at 6 hours (Figures 4, 5). The presence of MS^2^ product ion at *m/z* 362, which was estimated to be a dehydrated ion with degradation of the guanidine group, indicating that M4 has two primary amino groups (Table 2). Given these results, M4 was estimated to be a glucuronide of guanfacine, and its possible conjugation position was any position except for the amino groups.

### 3.10 | M5 metabolite

M5 was detected at the RT of approximately 17 min with *m/z* 422 in urine at 0–12 and 12–24 hours (Figure 5). M5 has the same molecular formula as M4. However, as *m/z* 362 in the MS^2^ spectrum was derived from M4 but not from M5, the fragmentations were different between M4 and M5 (Table 2). Therefore, M5 was estimated to be a glucuronide positional isomer of M4.

### 3.11 | M6 metabolite

M6 was detected at the RT of approximately 17 min with *m/z* 262 in urine at 0–12 and 12–24 hours (Figure 5). M6 has the same molecular formula as 3‐hydroxyguanfacine. Their fragmentations were equivalent (Table 2); however, they could be separated in the MS chromatogram (Figure 5). Therefore, M6 was estimated to be a positional isomer of 3‐hydroxyguanfacine.

### 3.12 | M7 metabolite

M7 was detected at the RT of approximately 21 min with *m/z* 422 in urine at 0–12 and 12–24 hours, and in plasma at 4, 6, 10 and 24 hours (Figures 4, 5). M7 has the same molecular formula as M4 and M5, though with different fragmentation patterns. The MS^2^ product ion at *m/z* 363 indicated that the possible conjugation position of glucuronic acid was the dichlorobenzyl moiety (Table 2). Given these results, M7 was estimated to be a glucuronide positional isomer of M4 and M5.

### 3.13 | M8 metabolite

M8 was detected at the RT of approximately 22 min with *m/z* 422 in urine at 0–12 and 12–24 hours and in plasma at 10 hours (Figure 5). M8 has the same molecular formula as M4, M5 and M7, though with different fragmentation patterns. The MS^2^ product ion at *m/z* 200 was estimated to be a fragment produced by conjugation of glucuronic acid and guanidine (Table 2). Therefore, the possible conjugation position of glucuronic acid was one of the amino groups. Given these results, M8 was estimated to be a glucuronide positional isomer of M4, M5 and M7.

### 3.14 | M9 metabolite

M9 was detected at the RT of approximately 25 min with *m/z* 398 in urine at 0–12 and 12–24 hours (Figure 5). The MS^2^ fragment ion at *m/z* 222 (−176) indicated that M9 was a glucuronide. Therefore, the molecular formula of the aglycone of M9 was estimated to be C_8_H_9_Cl_2_NO_2_. The molecular formula of the aglycone indicated that guanfacine was degraded at the guanidine group to amine, monooxidized and hydrogenated (+2H). The MS^2^ fragment ion at *m/z* 205 (−17) indicated that the aglycone of M9 has at least one primary amine group. Moreover, the MS^2^ fragment ion at *m/z* 194 was estimated to be a conjugate of glucuronic acid and amine (176 + 17 + 1). The MS^2^ fragment ion at *m/z* 159 was estimated to be a dehydrated ion of the aglycone (Table 2). Therefore, possible monooxidized and hydrogenated positions were on the dichlorobenzyl moiety. Given these results, M9 was estimated to be a glucuronide of a metabolite formed by monooxidation and hydrogenation of the dichlorobenzyl moiety and degradation of the guanidine group.

### 3.15 | Metabolic pathway

**Figure 6:**
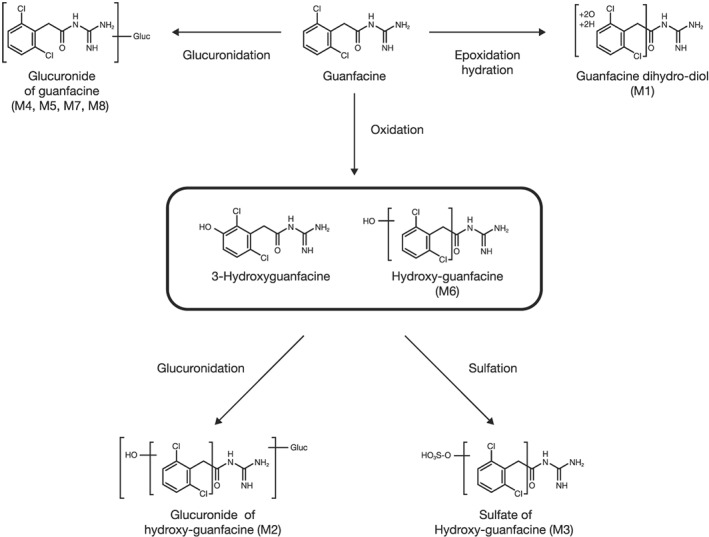
Proposed metabolic pathway of guanfacine

The proposed metabolic pathway of guanfacine is monooxidation (3‐hydroxyguanfacine and M6) at different positions on the dichlorobenzyl moiety, followed by glucuronide (M2) or sulfate (M3). Another minor pathway is glucuronide (M4, M5, M7 and M8) at different positions on guanfacine (Table 2 and Figure 6).

## 4 | DISCUSSION

Metabolite profiling and identification of guanfacine in humans has previously been conducted using urine samples (Kiechel, 1980), but not using plasma samples. Human plasma metabolite elucidation is increasingly important from the aspect of metabolite safety testing. The ICH M3 (R2) guideline recommends that, for nonclinical toxicity studies, ‘Nonclinical characterization of a human metabolite(s) is only warranted when that metabolite(s) is observed at exposures greater than 10% of total drug‐related exposure and at significantly greater levels in humans than the maximum exposure seen in the toxicity studies (ICH, 2012). The present study revealed that following oral administration of guanfacine, 3‐hydroxyguanfacine, M1 to M4, M7 and M8 were the plasma metabolites of guanfacine, with guanfacine being the most abundant component in plasma (from the MS signal intensity). M3 was found to be the most prominent plasma metabolite, followed by M2. Some specific human metabolites (glucuronide; M4, M7 and M8) were found in plasma samples, but only in trace quantities. There was no difference in composition ratio of the metabolites at each plasma sampling point. Subsequently, the exhaustive metabolic pathways of guanfacine were clarified as (1) monooxidation on the dichlorobenzyl moiety, followed by glucuronidation or sulfation, and (2) glucuronidation at different positions on guanfacine. Based on the present study, guanfacine is metabolically stable in humans. CYP3A4 is the predominant enzyme involved in the oxidative metabolism of guanfacine (Shire US Inc., 2018). Guanfacine does not appear to inhibit other major human CYP isozymes (CYP1A2, CYP2C8, CYP2C9, CYP2C19, CYP2D6 or CYP3A4/5) *in vitro* (Shire US Inc., 2018). In addition, the clinical study showed that a substantial increase (three‐fold) in guanfacine plasma exposure has been observed when guanfacine was co‐administered with ketoconazole, a strong CYP3A inhibitor (Shire Pharma Canada ULC, 2017). Conversely, a substantial decrease (by 70%) in guanfacine plasma exposure has been observed when guanfacine was co‐administered with rifampin, a strong CYP3A inducer (Shire Pharma Canada ULC, 2017).

There was a discrepancy between the composition ratio of each metabolite reported here and the results of clinical drug–drug interaction studies with co‐administered ketoconazole or rifampin (Shire US Inc, 2018).

In urine samples from the present study, unchanged guanfacine accounted for more than 90% of the total MS chromatogram peak area of the detected metabolites in each sample, and the metabolites accounted for less than 5%. There was no difference in the composition ratio of the metabolites at each urine sampling point. Meanwhile, in a human mass balance study after a single oral administration of ^14^C‐labeled guanfacine ([^14^C]‐guanfacine) at 3 mg, the metabolite in urine was investigated, and unchanged guanfacine accounted for 27.6% of radioactivity in urine samples (0–24 hours). The prominent metabolite in urine was reported to be the 3‐hydroxyguanfacine conjugated as either *O*‐glucuronide (34.5% of radioactivity in urine sample) or *O*‐sulfate (7.7%), and the metabolites were identified using HPLC, gas chromatography mass spectrometry and on‐line radioactivity detection (Kiechel, 1980). Thus, there was a difference in the composition ratio of guanfacine and its metabolites in human urine between the present study and the human mass balance study. In another study of a guanfacine‐related substance using LC–MS/MS and radio‐detector in human samples, it was suggested that the ionization efficiency of glucuronide and sulfate of 3‐hydroxyguanfacine was much lower (approximately 1/50) than that of guanfacine (unpublished observations: Shire Development LLC, a Takeda company data on file). The difference in the composition ratio seen here in urine might be caused by the analytical method (LC–MS/MS and radio‐detector) and ionization efficiency (guanfacine and its metabolites for LC–MS/MS). It was reported that there was a large difference in the ionization efficiency between unchanged form and Phase 2 metabolites (e.g. glucuronide) about many compounds at the drug discovery stage (Blanz, Williams, & Dayer, 2017). Similarly, there is a possibility that the composition ratio of the metabolites in human plasma was also underestimated in the present study, in which case these metabolites would be greater than 10% of the total drug‐related exposure in plasma, specifically M3 and M2 metabolites.

However, for drugs that have a daily administered dose of less than 10 mg, greater fractions of the drug‐related material (> 10% of total drug‐related exposure) might be more appropriate triggers for testing. Some metabolites are not of toxicological concern (e.g. most glutathione conjugates) and do not warrant testing (ICH, 2009). Most glucuronides are not of toxicological concern, except those that undergo chemical rearrangement (e.g. reactive acyl glucuronides) (ICH, 2012). Phase 2 conjugation reactions generally render a compound more water‐soluble and pharmacologically inactive, thereby eliminating the need for further evaluation (FDA, 2016). The clinical dose of guanfacine is 1–7 mg once daily (< 10 mg; Shire US Inc, 2018). The prominent metabolites (glucuronide and sulfate) detected in human plasma have also been observed in rats (glucuronide; 7% of radioactivity, sulfate; 34% of radioactivity in plasma sample at 0.5 hours post‐dose) and dogs (sulfate; 24% of radioactivity in plasma sample at 1 hour post‐dose) (unpublished observations: Shire Development LLC, a Takeda company data on file) and it was considered that the toxicity of these conjugations detected in the present study were not of concern. The nonclinical characterization of metabolites with an identified cause for concern (e.g. a unique human metabolite) should be considered on a case‐by‐case basis (ICH, 2009). From the results of the present study, no additional toxicity studies regarding guanfacine metabolites are likely to be necessary.

## 5 | CONCLUSIONS

Exhaustive metabolite analyses of guanfacine in plasma and urine were conducted using LC–MS/MS after repeated oral administration of guanfacine XR in healthy Japanese adult subjects. Unchanged guanfacine was the abundant component, and the M3 metabolite (a sulfate of hydroxy‐guanfacine) was the prominent metabolite in human plasma. The proposed main metabolic pathway of guanfacine is monooxidation on the dichlorobenzyl moiety, followed by glucuronidation or sulfation. Although there was a possibility that some metabolite levels were greater than 10% of the total drug‐related exposure in plasma, there was no need for further studies in animals to determine the potential toxicity of the metabolites, as the prominent metabolites in plasma were glucuronide and sulfate, in which toxicity is not of concern.

## CONFLICT OF INTEREST

Yuji Inoue, Hirotoshi Morita and Takushi Kanazu are employees of Shionogi & Co., Ltd. Kohei Nozawa is an employee of Sekisui Medical Co., Ltd. This study was funded by Shionogi & Co., Ltd., Shire, a Takeda company, and Shire International GmbH, a Takeda company.

## CLINICAL TRIAL REGISTRATION

The clinical trial in this study was not registered as it is not required to register clinical trials of Phase 1 studies (healthy adult volunteers) in Clinical Trials Information/JapicCTI.

## DATA SHARING AND DATA ACCESSIBILITY

The content has not been submitted for publication elsewhere except as a brief abstract in the proceedings of a scientific meeting.
